# Characterization of Murine Thymic Stromal-Cell Lines Immortalized by Temperature-Sensitive Simian Virus 40 Large T or Adenovirus 5 E1a

**DOI:** 10.1155/1991/14636

**Published:** 1991

**Authors:** Lena Larsson, Emma Timms, Kenneth Blight, Deborah E. Restall, Parmjit S. Jat, Amanda G. Fisher

**Affiliations:** 1 ICRF Human Tumour Immunology Group University College and Middlesex School of Medicine 91 Riding House Street London W1P 8BT UK; 2 ICRF Tumour Immunology Group University College London Gower Street London UK; 3 ICRF Electron Microscopy Unit Lincolns Inn Field London UK; 4 Ludwig Institute for Cancer Research 91 Riding House Street London W1P 8BT UK

## Abstract

The heterogeneity of thymic stromal cells is probably related to their role in providing different microenvironments where T cells can develop. We have immortalized thymic stromal elements using recombinant retroviral constructs containing a temperature-sensitive simian virus 40 (SV40tsA58) large-T antigen gene or the adenovirus 5 E1a region linked to the gene coding for resistance to G418. Cell lines containing the thermolabile large T antigen encoded by SV40 proliferate at the permissive temperature of 33°C and arrest growth when transferred to the nonpermissive temperature of 39°C. At the nonpermissive temperature, ts-derived cell lines are shown to alter their phenotype but remain metabolically active, as indicated by the inducible expression of class I and class II MHC antigens. Here we describe the generation of a total of 84 thymic stromal-cell lines, many of which show distinct morphologic, phenotypic, and functional properties consistent with fibroblastoid, epithelial, or monocytoid origins. Several E1a and SV40tsA58-derived cell lines generated exhibit the epithelial characteristic of desmosome formation and, in addition, two of these lines (15.5 and 15.18) form multicellular complexes (rosettes) when incubated with unfractionated thymocytes from syngeneic mice. A single line (14.5) displays very strong nonspecific esterase activity, suggesting it may represent a macrophagelike cell type. We describe the generation of stromal cell lines with different properties, which is consistent with the heterogeneity found in the thymic microenvironment. In addition to documenting this diversity, these cell lines may be useful tools for studying T-cell development *in vitro* and give access to model systems in which stromal-thymocyte interactions can be examined.


*Developmental Immunology*, 1991, Vol. 1, pp. 279–293
Reprints available directly from the publisher
Photocopying permitted by license only

© 1991 Harwood Academic Publishers GmbH
Printed in the United Kingdom

### Characterization of Murine Thymic Stromal-Cell Lines Immortalized by Temperature-Sensitive Simian Virus 40 Large T or Adenovirus 5 Ela

LENA LARSSON,*† EMMA TIMMS,‡ KENNETH BLIGHT, DEBORAH E. RESTALL,† PARMJIT S. JAT,§ and AMANDA G. FISHER†

*†ICRF Human Tumour Immunology Group, University College and Middlesex School of Medicine, 91 Riding House Street, London W1P 8BT, U.K.*
*‡ICRF Tumour Immunology Group, University College London, Gower Street, London, U.K.*
*ICRF Electron Microscopy Unit, Lincolns Inn Field, London, U.K.*
*§Ludwig Institute for Cancer Research, 91 Riding House Street, London W1P 8BT, U.K.*

The heterogeneity of thymic stromal cells is probably related to their role in providing different microenvironments where T cells can develop. We have immortalized thymic stromal elements using recombinant retroviral constructs containing a temperature-sensitive simian virus 40 (SV40tsA58) large-T antigen gene or the adenovirus 5 Ela region linked to the gene coding for resistance to G418. Cell lines containing the thermolabile large T antigen encoded by SV40 proliferate at the permissive temperature of 33°C and arrest growth when transferred to the nonpermissive temperature of 39°C. At the nonpermissive temperature, ts-derived cell lines are shown to alter their phenotype but remain metabolically active, as indicated by the inducible expression of class I and class II MHC antigens. Here we describe the generation of a total of 84 thymic stromal-cell lines, many of which show distinct morphologic, phenotypic, and functional properties consistent with fibroblastoid, epithelial, or monocytoid origins. Several Ela and SV40tsA58-derived cell lines generated exhibit the epithelial characteristic of desmosome formation and, in addition, two of these lines (15.5 and 15.18) form multicellular complexes (rosettes) when incubated with unfractionated thymocytes from syngeneic mice. A single line (14.5) displays very strong nonspecific esterase activity, suggesting it may represent a macrophagelike cell type. We describe the generation of stromal cell lines with different properties, which is consistent with the heterogeneity found in the thymic microenvironment. In addition to documenting this diversity, these cell lines may be useful tools for studying T-cell development *in vitro* and give access to model systems in which stromal-thymocyte interactions can be examined.

KEYWORDS: Thymus, stromal-cell lines, temperature-sensitive SV40 large T, Ela.

### INTRODUCTION

The thymic microenvironment is composed of multiple cell types of nonhematopoietic and hematopoietic origin that support stem-cell proliferation and T-cell differentiation (Moore and Owen, 1967). Direct contact between stromal elements and thymocytes is believed to be crucial for the development of functionally mature, antigen-specific and MHC-restricted T-lymphocytes (Owen and Ritter, 1969; Stutman, 1978). The nature of these cell–cell interactions and also the effect of soluble factors known to be involved at different stages of T-cell development are not fully understood. Previous studies have suggested that (i) contact between epithelial cells and progenitors involves specific cell–surface interactions (such as CD2 and its natural ligand LFA-3) (Selvaraj et al., 1987; Vollger et al., 1987), (ii) inductive interactions are probably mediated by physiological factors/receptors (such as IL-2

*Corresponding author.

and its receptors) (Ceredig et al., 1985; Jenkinson et al., 1987), (iii) expression of class I and class II MHC antigens on the thymic stroma lead to the acquisition of MHC restriction and self-tolerance by maturing T cells (Zinkernagel, 1982; Lo and Sprent, 1986; von Boehmer and Hafen, 1986), and (iv) that these interactions can ultimately lead to the expression of a more differentiated phenotype by progeny cells, such as the progressive rearrangement of T-cell receptor genes (TCR) (Davis and Bjorkman, 1988) and/or the expression of Thy-1, CD3, CD4, and CD8 (Borst et al., 1983; Fowlkes et al., 1985; Smith, 1987).

An understanding of the events that control T-cell development in the thymus has been hampered by the lack of in vitro systems. One method that has been widely used is the mouse thymic foetal organ culture system (van Ewijk et al., 1982). This model has proved to be very useful for studying lymphopoiesis, but due to its complexity, it has been difficult to assess the specific roles of the various types of cells in the thymic microenvironment. In an attempt to understand the possible role of different microenvironments within the thymus, we have attempted to immortalize different stromal elements using a temperature-sensitive mutant of SV40 large-T antigen (Tegtmeyer, 1975; Jat and Sharp, 1989) or E1a constructs (Roberts et al., 1985). We report the isolation of 84 cell lines following retroviral infections of primary embryonic stromal cultures or thymocyte rosette/thymic nurse cell (T-ROS/TNC) cultures isolated from adult thymuses.

### RESULTS

### Immortalization of Thymic Stromal Cells

Eighty-four thymic stromal cell lines were isolated by infecting primary cultures of foetal thymus with recombinant retroviruses SV40tsA58 (Tegtmeyer, 1975; Jat and Sharp, 1989) or E1a 12S (Roberts et al., 1985), and selecting colonies resistant to G418 (500 $\mu$g/ml) (Davies and Jinenez, 1980). In the first set of experiments (see Table 1) most clones isolated by infection with SV40tsA58 (49 of 72) had a fibroblastic appearance, judged by morphology and strong vimentin expression. Two such cell lines (designated 5.10 and 7.5) were cloned and selected for further study as representatives of this large stromal-cell group. Approximately one-third of cell lines obtained from Sv40tsA58 infections (23 of 72) had a markedly different morphology, with a polyg-

TABLE 1
Murine Thymic Stromal-Cell Lines Isolated by Retroviral Infection

| Retrovirus used for infection | Type of culture infected | Morphology of cells derived | Number of clones/ total number isolated | Designation of clones selected for further study |
|---|---|---|---|---|
| Zip SVlsa58 | Primary embryonic thymic stromal | Fibroblastoid | 49/72 | 5.10, 7.5 |
| Zip SVlsa58 | Primary embryonic thymic stromal | Polygonal | 23/72 | 6.10[a] |
| Zip E1a 12S | Primary embryonic thymic stromal | Fibroblastoid | 3/7 | none |
| Zip E1a 12S | Primary embryonic thymic stromal | Cobblestone | 1/7 | 8.40[b] |
| Zip E1a 12S | Primary embryonic thymic stromal | "Fried egg" | 3/7 | 14.5 |
| Zip E1a 12S | Adult thymic T-ROS/TNC[c] fractions | Polygonal | 2/5 | 15.5, 15.18 |

[a]A subclone of 6.10, designated 6.10[13], in which cells displayed a uniform mosaic appearance was also derived and selected for subsequent examination.
[b]Derived from mouse strain AKR/Icrf; all other selected clones from BALB/c.
[c]Thymocyte rosettes/thymic nurse cell.

onal outline. Among this cell type, clone 6.10 was selected for further study and a subclone that displayed a mosaiclike morphology (6.10 subclone 13) was also isolated. Infection with E1a 12S virus yielded a more limited panel of seven cell lines in which three morphological cell types could be distinguished. Cell lines with a fibroblastic appearance (3 of 7) were not studied further. Two clones (8.40 and 14.5) morphologically distinct from fibroblasts were selected for detailed characterization.

A second series of experiments was performed in an attempt to generate stromal-cell lines representing epithelial, dendritic, and macrophage elements. To avoid the repeated generation of fibroblastlike cell lines, thymocyte rosettes (T-ROS; medullary dendritic or cortical macrophages with thymocytes bound), and thymic nurse cells (TNC; lymphoepithelial complexes containing cortical epithelium and fused thymocytes) were isolated from adult thymus by a series of enzymatic digests and separations (Kyewski et al., 1982) (see Materials and Methods). These enriched populations of cells were used as targets for virus infection, and five additional polygonal cell lines were obtained with E1a 12S of which two (15.5 and 15.18) were expanded, cloned, and retained for further study.

### Temperature-Dependent Growth and Differentiation of Cell Lines Derived from SV40tsA58 Infection

The rationale for using SV40tsA58 as an immortalizing agent to generate thymic stromal-cell lines was that cells might be clonally expanded at 33°C (SV40 large T permissive temperature) and yet stop proliferating and adopt a more "normal", differentiated phenotype at 39°C (at which temperature SV40 large T is nonfunctional). In view of this, the growth of cell lines 5.10, 7.5, and 6.10 at 33°C and 39°C was studied in detail (see Fig. 1A). A well-characterized rat fibroblastic line (tsa 8) obtained from infection of primary skin cultures with SV40tsA58 (Jat and Sharp, 1989) was used as a control in these studies, as were two E1a 12S-derived clones 8.40 and 14.5. Cells were initially plated at a density of $2\times10^4$ cells/60 mm dish and grown either at 33°C or 39°C (plus 37°C for clones 8.40 and 14.5). As shown in Fig. 1A, clones tsa 8, 5.10, 7.5, and 6.10 grew well at 33°C (with a doubling time of approximately 30–36 hr), but grew poorly or not at all when maintained at the higher temperature (39°C). In contrast, clones obtained from E1a 12S infection (8.40 and 14.5) grew extremely slowly at 33°C, but grew well at 37°C and 39°C. These data suggest that the immortalization of clones 5.10, 7.5, and 6.10 is dependent on a temperature-sensitive element (SV40 large T). This was verified by direct staining of cells at 33°C and 39°C with a monoclonal antibody (mAb) to SV40 large-T product (PAb 412). As shown in Fig. 1B (lower panels), strong nuclear staining was observed when these cells were grown at 33°C, but not after being maintained at the nonpermissive temperature of 39°C. Concomitant with the change in SV40 large-T expression at 39°C, clones 5.10, 7.5, and 6.10 were also observed to undergo a number of other changes. Most notably the cytoplasm to nucleus ratio increased, many cells adopted a multinucleated appearance (perhaps indicating a general problem in successfully completing cell division), and the cells became contact-inhibited (see upper right panel of Fig. 1B). Taken together, these findings suggest that clones 5.10, 7.5, and 6.10 are temperature-sensitive and that switching to a nonpermissive temperature not only leads to cessation of growth, but also to alterations in their properties consistent with a more "normal" less-transformed phenotype.

### Properties and Lineages of Isolated Stromal-Cell Lines

To determine whether thymus-derived stromal cells generated in this study were of epithelial, fibroblastoid, dendritic, or macrophage origin, a series of experiments was carried out. In the first study, all cell lines were tested for their ability to express the intermediate filaments (IF) keratin and vimentin. Intermediate filaments are a heterogenous group of cytoskeletal proteins whose expression and function are dependent on the type and differentiated state of the cell (Lazarides, 1982). All cell lines expressed detectable levels of vimentin and some variation of both intensity and pattern of intracellular staining was observed between cells grown at different temperatures (see Table 2 and Fig. 1B). No staining was observed using two different antibodies to keratin (the mAb; LE61 and a polyclonal antiserum raised in rabbit). This lack of reactivity did not rule out the possibility that

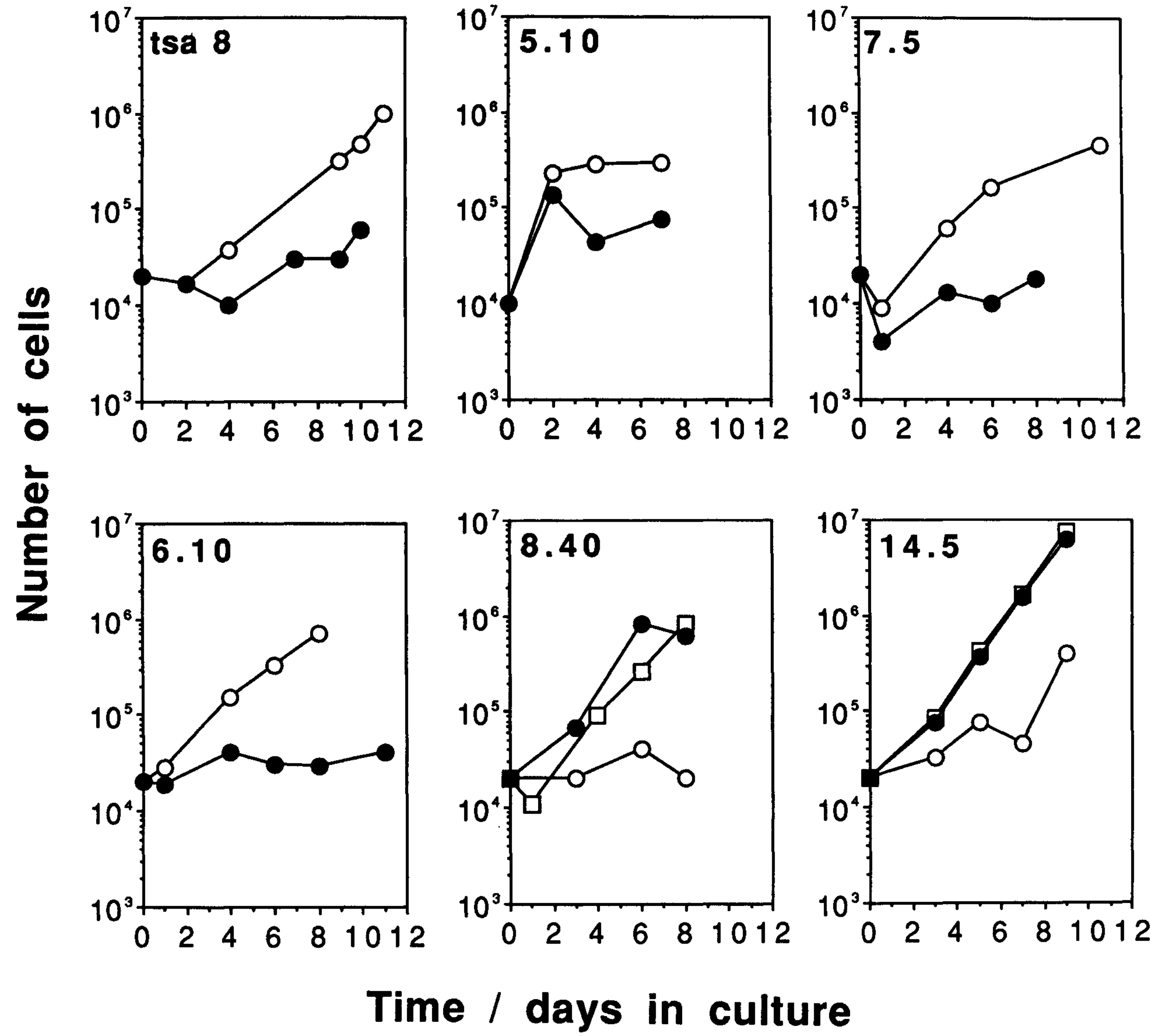

FIGURE 1A. Growth kinetics at permissive and nonpermissive temperatures. Three SV40ts clones (5.10, 7.5, and 6.10), two E1a clones (8.40 and 14.5), and the established ts clone tsa 8 (used as a control) were examined. Cells were harvested and counted at different time points. Values are means of two independent experiments, where each point represents counts on duplicate cultures, 33°C (○), 37°C (□), and 39°C (●).

some of our stromal-cell panel might be epithelial since epithelial cells undergoing differentiation and epithelial cells in primary culture have been shown to alter their IF expression in some cases (Kim et al., 1987; Ben-Zèev 1984).

In a second set of experiments, electron microscopy studies were performed to look for desmosomal bodies, which are characteristic of epithelial cells (Farquhar and Palade, 1963). As illustrated in Table 2 and Figs. 2D–2F, desmosomal junctions were readily identified in preparations from four cell lines: 6.10, 8.40, 15.5, and 15.18. An EM image of whole 15.5 cells demonstrates that the cells form junctions at the adjacent cellular membranes (Fig. 3). Fine tonofilament fibers were found along the cell membranes in this cell line. Although bundles of tonofilaments cannot be seen here, they were present in other cell preparations. Furthermore, cell organelles such as mitochondria and lysosomes were present at abundant levels. These results together with their morphological appearance,

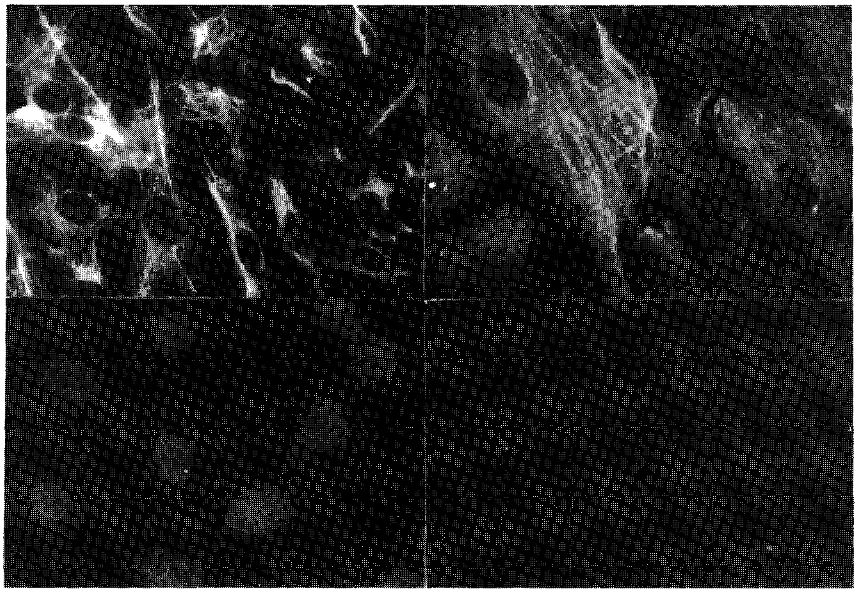

FIGURE 1B. Temperature dependence of ts clones grown at 33°C and 39°C. Examination of vimentin and SV40 large-T expression on SV40tsA58 clones by immunofluorescence staining on acetone:methanol fixed cells.

TABLE 2
Characterization of Selected Clones

| Clone | Vimentin expression[a] | Keratin expression[b] | Desmosome formation[c] | Nonspecific esterase activity[d] | Rosette formation[e] (%) | Proposed cell lineage |
|---|---|---|---|---|---|---|
| 5.10 | +++ | – | – | – | 3 | Fibroblast |
| 6.10 | ++ | – | + | – | 14 | Epithelial |
| 7.5 | +++ | – | – | – | 16 | Fibroblast |
| 8.40 | + | – | + | + | 4 | Uncertain |
| 14.5 | + | – | – | ++ | 5 | Uncertain |
| 15.5 | + | – | + | – | >80 | Epithelial |
| 15.18 | + | – | +/– | +/– | >70 | Epithelial |
| tsa 8 | + | – | NT[g] | – | 2 | Rat embryonic fibroblast[f] |
| Caski | + | + | NT | NT | NT | Human epithelial[h] |

[a]Vimentin expression was tested using a goat antimouse vimentin antibody (ICN) and FITC-conjugated second-layer antibody.
[b]Keratin expression revealed by immunofluorescence staining on acetone:methanol fixed cells using the antibody LE61 and FITC-conjugated antimouse reagent.
[c]Investigated by electron microscopy of 2.5% glutaraldehyde fixed, confluent cultures.
[d]Tested using standard techniques (34), monocytoid cells were used as positive control for staining.
[e]Stromal cells and unfractionated adult thymocytes (1:12) were mixed, centrifuged at 4°C, and the number of rosettes counted using a hemocytometer (>3 thymocytes bound was scored as a rosette). Values are means of 2–5 independent experiments.
[f]An established cell line from primary skin cultures, generated from a SVtsA58 infection (16).
[g]NT=not tested.
[h]A human squamous epithelial-cell line derived from a carcinoma of the cervix (35).

which differs from putative fibroblast lines (5.10 and 7.5), suggest that these cell types are of epithelial origin.

In addition to these studies, a third set of experiments was carried out in which cell lines were stained with a panel of antibodies and assessed for nonspecific esterase activity. Two mAb to mouse thymic epithelial cells (TEC) (ER-TR4; recognizing cortical epithelium and ER-TR5; recognizing medullary epithelium) (van Vliet et al., 1984) were unreactive in all cases tested. Epithelial heterogeneity has been documented

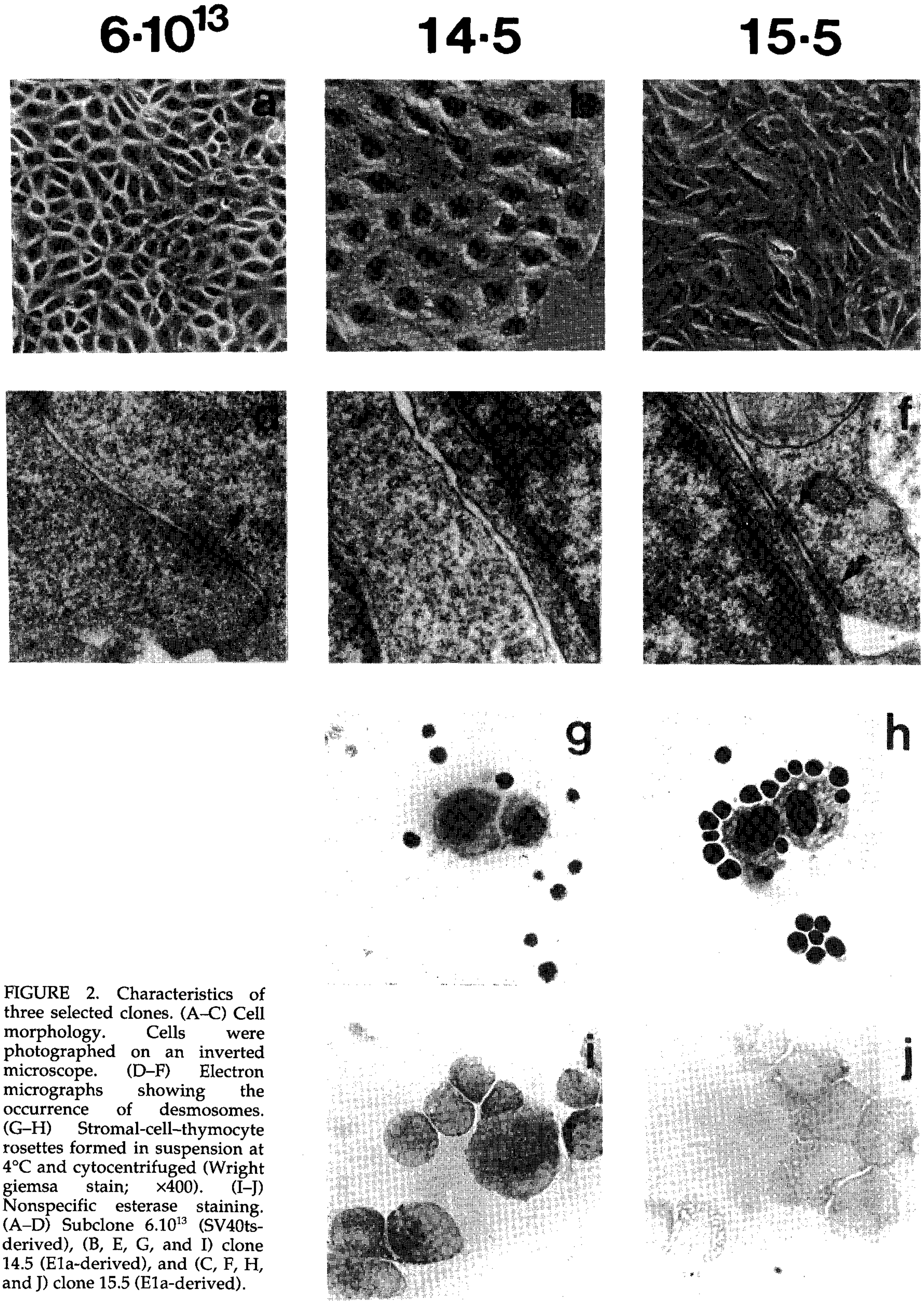

FIGURE 2. Characteristics of three selected clones. (A–C) Cell morphology. Cells were photographed on an inverted microscope. (D–F) Electron micrographs showing the occurrence of desmosomes. (G–H) Stromal-cell–thymocyte rosettes formed in suspension at 4°C and cytocentrifuged (Wright giemsa stain; ×400). (I–J) Nonspecific esterase staining. (A–D) Subclone $6.10^{13}$ (SV40ts-derived), (B, E, G, and I) clone 14.5 (E1a-derived), and (C, F, H, and J) clone 15.5 (E1a-derived).

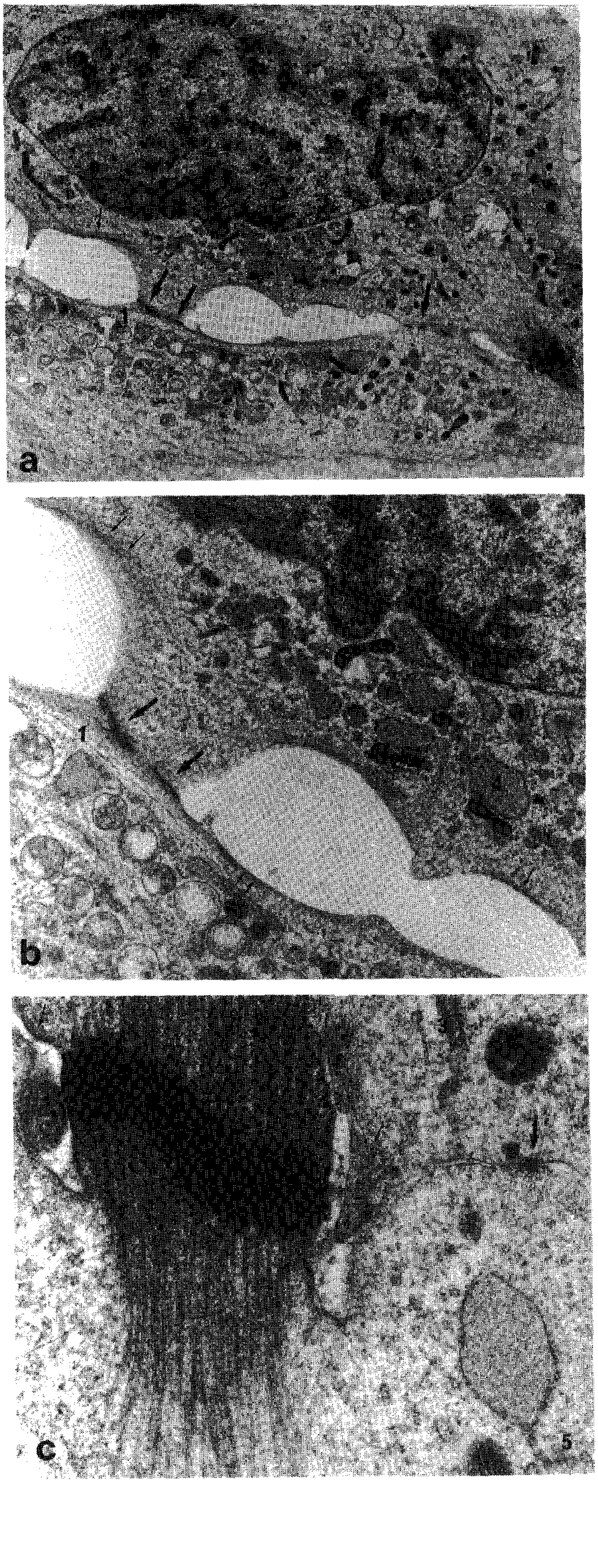

FIGURE 3. Transmission electron micrographs of 15.5 cells. In (A), (B), and (C), desmosomelike junctions are present at the cell–cell contact points (thick arrows). Fine tonofilament fibers are seen close to the cellular membranes and in the cytoplasm (thin arrows). Cellular organelles are marked (1) microtubules, (2) mitochondria, (3) rough endoplasmic reticulum, (4) lysosomes, and (5) ribosomes. The mitotic spindle (midbody) in (A) and (C) is marked (6). (B) and (C) represent low-power micrographs of (A).

within the medulla from the staining pattern obtained by two L-fucose-binding lectins (Farr and Anderson, 1985). Ulex europeus agglutinin (UEA) and tetragonolobus purpureas agglutinin (TPA) have shown to have specificity for reticular epithelial cells and Hassall's corpuscles in the medulla, respectively. All cell lines were negative for these lectins and this result in conjunction with the negative result by the ER-TR mAbs therefore failed to clarify a possible epithelial origin. Next, a number of antibodies to haematopoietic stromal components were tested, NLDL-145 and MIDC-8 (Kraak et al., 1986; Breel et al., 1987) (recognizing dendritic cells), Mac-1 (reacting with cells of monocyte/macrophage lineage) (Springer et al., 1979), and M193 (Springer et al., 1978) (an antibody to the leukocyte common antigen CD45). These antibodies were also unreactive with all cell lines tested (data not shown). The T-lymphocyte marker Thy-1 has been shown to be present on a variety of stromal-cell types of mouse, including fibroblasts and neural cells (McKenzie and Potter, 1979), epidermal cells (Konig et al., 1987), stromal-cell lines of bone marrow and spleen (Pietrangeli et al., 1988), and thymic epithelium (Tucek and Boyd, 1990). To examine whether our clones express this antigen, cells were stained in suspension using antibodies to Thy-1 (Ledbetter and Herzenberg, 1979; Marshak-Rothstein et al., 1979) and analyzed on a FACScan. One line, 15.5, contained 50–60% Thy-$1^+$ cells and the positive population exhibited high levels of expression (data not shown). Nonspecific esterase activity, which appears to be associated particularly with cells of monocyte/macrophage lineage, was clearly demonstrated with clone 14.5 (see Table 2 and Fig. 2I), which is somewhat puzzling in view of our failure to identify Mac-1 or CD45-positive cells.

Since cell lines 15.5 and 15.18 (tentatively assigned an epithelial lineage) were originally isolated from T-ROS/TNC cultures, containing multicellular complexes between stromal cells and thymocytes, it was of interest to assess whether these cells still retain this functional capacity after immortalization and cloning. In a fourth set of experiments, unfractionated adult thymocytes, which consist largely of immature double-positive ($CD4^+CD8^+$) T cells (data not shown), were used as targets in a rosette assay. As shown in Table 2 and illustrated in Figs. 2G and 2H, five of eight stromal lines showed little or no rosetting capacity (above that of the fibroblast line tsa 8, used as a control). E1a-derived lines (15.5 and 15.18) formed approximately 80% and 70% rosettes, respectively. Since both 15.5 and 15.18 have been shown to form desmosomes, these data suggest that these cells may represent cortical epithelial cells, which are believed to form complexes with thymocytes *in vivo*.

### Expression of MHC Class I and Class II Antigens on Murine Thymic Stromal Cell Lines

Expression of MHC class I and II is believed to be crucial for the generation of mature $CD4^+$ and $CD8^+$ T cells in the thymus (Wekerle et al., 1980b; Doyle and Strominger, 1987; Marusic-Gale et al., 1988). Therefore, it was of interest to examine the level of expression of these molecules on our stromal lines and to determine whether the expression could be influenced by IFN$\gamma$ and/or by maintaining the cells at 33°C or 39°C. In these studies, stromal cells were cultured in IMDM/FCS supplemented with 5, 50, or 500 U/ml IFN$\gamma$ and class I and II expression was monitored using antibodies HB24 and TIB120, respectively. All three cell lines derived from infection with SVtsA58 (5.10, 6.10, and 7.5) express MHC class I in the presence of IFN$\gamma$ (results of clone 5.10 and 6.10 are shown in Fig. 4, top panels). However, their response to changing temperature differs. Class I expression on 5.10 in the absence of IFN$\gamma$ is unaffected by temperature, whereas 6.10 more readily expresses class I when maintained at 39°C, when SV40 large T is nonfunctional. In our experiments, clone 5.10 did not express class II antigens at either 33°C or 39°C and was not sensitive to induction by IFN$\gamma$. On the other hand, clone 6.10, which also failed to express class II at the cell surface at the permissive temperature of 33°C, could be induced with IFN$\gamma$ to express class II at the nonpermissive temperature of 39°C. These variations in MHC class I and class II expression and inducibility of ts-clones were also reflected in the panel of cells generated by infection with retroviruses containing E1a (see lower panels of Fig. 4). Cell line 14.5 expressed class I poorly and required activation by IFN$\gamma$. In contrast, cell line 15.5 expressed class I constitutively at higher levels (cell lines 8.40 and 15.18 showed similar staining pattern as 15.5; data not shown). Both cell types (14.5 and 15.5) appear equally dependent on the induction of

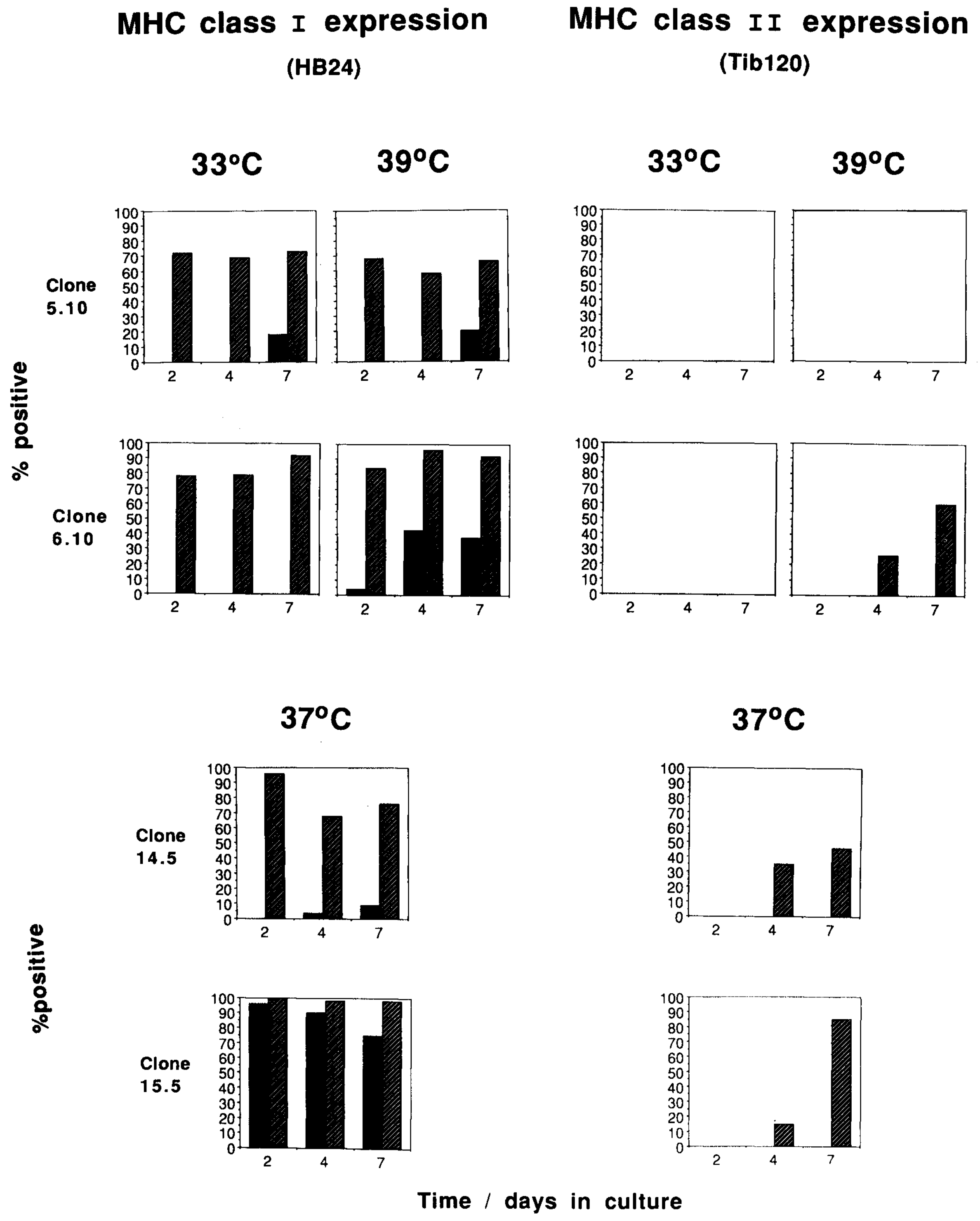

FIGURE 4. Expression of MHC class I and II antigens on stromal-cell lines under the influence of temperature switching and/or IFNγ. The percentage of cells expressing class I and II histocompatibility is shown for SV40ts clones (5.10 and 6.10) and E1a clones (14.5 and 15.5). Expression was measured following culture at 33°C or 39°C and monitored at different days after treatment with IFNγ (500 U/ml) (▨) or medium alone (■). Antibodies HB24 and TIB120 (and FITC-conjugated second-step reagents) were used to measure MHC class I and class II expression, respectively. Results from a single experiment are shown that are representative of multiple (3) independent experiments.

IFN$\gamma$ for the expression of class II at the cell surface (Fig. 4, bottom right panels). Clone 15.18 expressed class II after induction by IFN$\gamma$, similar to 15.5; in contrast, clone 8.40 was unable to express class II under any conditions tested (data not shown).

### DISCUSSION

The thymus is composed of a variety of stromal elements including cells of nonhematopoietic origin (fibroblasts and epithelial cells) and cells of hematopoietic lineage (macrophages and dendritic cells). These stromal cells provide different microenvironments in which T-cell precursors can develop into functionally mature T cells. Therefore, in order to understand how T-cell lymphopoiesis is regulated, it is important to document the specific role of each cell type. One approach to study the complex architecture of the microenvironment has been to raise mAb to different stromal elements. A large panel of reagents have been produced and cataloged into groups, recognizing distinct areas of the thymus (reviewed by Brekelmans and van Ewijk, 1990). Another approach to study the function of the microenvironment has been to isolate stromal cells and generate corresponding cell lines. This has been used in human, rat, and mouse systems (Itoh et al., 1981; Beardsley et al., 1983; Mitzutani et al., 1987). A problem with this approach is obtaining cell lines with functional properties that reflect their normal counterparts *in vivo*, for example, stromal cells that assist T cells to mature and differentiate. At present, the number of functional stromal-cell lines is small. To circumvent such limitations, we tried to generate cell lines using a temperature-sensitive mutant of SV40 large T. In addition, the transforming agent E1a was also employed to overcome a possible limitation in cell populations susceptible to the immortalizing effects of SV40 large T. Moreover, to increase the possibility of generating cell lines useful for *in vitro* reconstitution of *in vivo* events, cells were preselected prior to immortalization, on the basis of certain functional criteria (capacity to form multicellular complexes *in vivo*).

From several retroviral infections, 84 lines were generated, maintained, and characterized in terms of their morphology and intermediate filament expression. From that large group of cell lines, seven clones of a panel were characterized in more detail and found to resemble stromal cells of different origins judged by their morphology and phenotype. One E1a-derived line, 14.5, had a distinct morphology; the cells appeared very flat in culture ("fried-egg shape") and had strong nonspecific esterase activity (indicative of cells of the monocyte/macrophage lineage). That we were unsuccessful in finding Mac-1 or CD45, expressing cells requires further investigation in order to identify the origin of 14.5. Clones 5.10 and 7.5 (derived from an SV40tsA58 infection) appeared fibroblastoid according to their elongated morphology, the absence of desmosomes, and their lack of reactivity with antibodies recognizing cells of macrophage, dendritic, or epithelial origin. Another ts-derived cell line, 6.10, also failed to stain with any of the lineage-specific markers tested, but in contrast to 5.10 and 7.5, formed desmosomes, suggesting it may be an epithelial cell type. Desmosomes were also found among three E1a-derived lines, 8.40, 15.5, and 15.18. Clones 15.5 and 15.18 (isolated from a T-ROS/TNC culture) retained the capacity to form multicellular complexes with adult unfractionated thymocytes *in vitro*. In contrast, clones 6.10 and 8.40 (derived from primary culture of embryonic thymus) did not have this capacity, suggesting that the origin (embryonic or adult) and/or the type of culture they were isolated from (primary stromal or T-ROS/TNC) may influence the phenotypic range of cells that are immortalized.

TEC can be divded into different subpopulations, depending on location in the thymus and reactivity with defined monoclonal antibodies. Therefore, it was of interest to establish whether clones shown to form desmosomes (6.10, 8.40, 15.5, and 15.18), but with different morphology and functional ability, resembled epithelial cells of different origin. In order to do so, we adopted the criteria for TEC lines proposed by Brekelmans and van Ewijk (1990): (1) presence of desmosomes and tonofilaments, studied by EM; (2) detection of cytokeratin with mAb; (3) detection of TEC-specific antigens with mAb; and (4) absence of antigens specific for macrophages, dendritic cells, and fibroblasts, identified with mAb. Our isolated clones tentatively proposed to be of epithelial origin only fulfill two of these four criteria, requirements (1) and (4). However, both 15.5 and 15.18 form rosettes with thymocytes in which the majority of bound cells express both CD4 and CD8 (>93%) (data not shown). This phenotype is typical of cortical thymocytes (Fink et al., 1984; Kyewski et al., 1987), which might suggest that these cell lines (15.5 and 15.18) resemble cortical epithelial cells. The failure to demonstrate ER-TR4$^+$ ER-TR5$^-$ cells (a mAb-defined phenotype, indicative of cortex-derived epithelial cells) and cytokeratin expression by 15.5 and 15.18 needs clarification. However, it is perhaps worth noting that cytokeratin expression is known to be heterogenous and dependent on the differentiated state of the cell (Lazarides, 1982; Ben-Zeev, 1984; Kim et al., 1987), which may in part explain our results. Furthermore, long-term culturing may also result in decreased or lost expression of certain antigens, as have been reported by Cattermole et al. (1989). In order to establish whether the lack of reactivity with a number of stromal-cell specific markers is a result of immortalization and/or long-term culturing, conventional lines could perhaps be used as targets for infection. This kind of study would be valuable to investigate what phenotypic changes occur after a retrovirus has been introduced into the cell and in addition, how well the immortalized and nonimmortalized cells represent their counterparts *in vivo*.

Several investigators have reported the generation of murine stromal-cell lines including those of epithelial origin: TEP1 (Beardsley et al., 1983); E5 (Potworowski et al., 1986); TE-71 and TE-75 (Farr et al., 1989); and St3 (Brightman et al., 1989). Many of these lines display a medullary phenotype according to their reactivity with ER-TR5 (and failure to react with ER-TR4). All these cell lines have been established by continuous culture and cloning without the use of immortalizing agents. It seems that this method is sufficient for generating epithelial cell lines. However, certain cell populations within the thymic microenvironment may have a limited proliferation capacity and, therefore, cannot be generated in this way. To overcome this limitation, it seems attractive to use transforming agents in order to obtain cell lines with different characteristics. Our data show that embryonic fibroblasts are the predominant targets for SV40 transformation in our system, whereas E1a appears to contain the capacity to immortalize epithelial- and macrophagelike cell types. In this context, it is interesting to note that recombinant retroviruses containing the v-*myc* and v-Ha-*ras* oncogenes have been shown to generate a different panel of stromal-cell lines from embryonic thymus (Cattermole et al., 1989). Here, a large group of adherent cell lines, most likely representing cells from the macrophage/dendritic cell compartment (concluded by the presence of Mac-1, Fc receptors, and class II) was established. A second group of adherent stromal lines was also established. This group showed no expression of macrophage/dendritic-cell and epithelial-cell markers tested. Taken together, in order to completely reconstruct the thymic microenvironment *in vitro*, it may be necessary to use a variety of immortalizing agents.

Few stromal-cell lines with functional capacity, allowing T-cell differentiation, have been reported. Recently, Palacios et al. (1989) reported a mouse TEC line capable of mediating differentiation of PRO-T lymphocyte clones into TCR/CD3-expressing cells. Another report by Brightman et al. (1989) shows that the stromal-cell line St3 can induce expression of Thy-1 and CD4 by a T-lymphoid cell line. Two mouse thymic stromal-cell lines (MRL-104.8a and TEL-2) hve been described, which have been claimed to induce selective elimination of immature double-positive (CD4$^+$CD8$^+$) thymocytes and a T-cell clone, respectively (Kosaka et al., 1989; Nakashima et al., 1990). No functional properties of the possible epithelial lines designated TG and produced by v-*myc* and v-Ha-*ras* transformation have been reported (Cattermole et al., 1989). It is believed that cortical epithelial cells are involved in positive selection for MHC following contact with immature thymocytes (Benoist and Mathis, 1989; Berg et al., 1989). Since clones 15.5 and 15.18 express MHC class I and will express class II afrer induction by IFN$\gamma$, these cell lines may prove useful for studying the requirements for positive selection and the signals required for differentiation or cell death in the thymus. Furthermore, these cells will allow us to study multicellular complex formation at the molecular/biochemical level.

In this study, 72 SV40tsA58-derived cell lines were isolated. By switching the cells to the nonpermissive temperature of 39°C, we hoped to observe a change in phenotype to a more "normal differentiated" cell. Our findings, that clone 6.10 will only express MHC class I after culture at the nonpermissive temperature of 39°C (unless supplemented with IFN$\gamma$), and that class II is only inducible at 39°C (after addition of IFN$\gamma$), suggest that cells cultured without the influence of large T do adopt a more normal phenotype. The ts lines described here also provide an opportunity to search for genes corresponding to novel thymic growth factors (by subtractive cDNA approaches at 33°C versus 39°C). An extension of this approach is to introduce the ts mutant into the germline of mice. This transgenic model system, pioneered by Jat et al. (1990), yields a variety of interesting conditionally immortalized stromal cells. These combined approaches suggest that the use of SV40ts large T and E1a as immortalizing agents may have widespread application for studying T-cell differentiation *in vitro*.

### MATERIALS AND METHODS

### Animals

BALB/c and AKR/Icrf mice were obtained from the ICRF breeding unit. Foetuses were removed from pregnant mice 14 days after observation of a vaginal plug.

### Cells and Cell Culture

Foetal thymic lobes, isolated at the fourteenth day of gestation, were cultured on Nuclepore (Sterilin, Feltham, U.K.) filters floating in Iscove's modified Dulbecco's medium (IMDM) supplemented with 10% foetal calf serum (FCS) and antibiotics (Gibco) (IMDM/FCS) (van Ewijk et al., 1982) for 0 to 6 days. Primary embryonic stromal cultures were established by covering disrupted foetal thymic lobes with small glass coverslips in a 60-mm petri dish containing IMDM/FCS (Singer et al., 1985). Thymocyte rosette/thymic nurse cell (T-ROS/TNC) cultures were prepared by enzyme digestions of adult thymuses, as previously described (Kyewski et al., 1982). Adult thymuses (15–20), obtained form BALB/c mice, were minced with sharp scissors and incubated in 10 ml of RPMI-1640 with 25 mM Hepes (RT), supplemented with 3% FCS for 15 min at room temperature (RT) during which time the fragments were gently agitated and free thymocytes were removed and discarded. The remaining thymic fragments were then incubated in 7 ml of the same medium containing collagenase (Worthington Biochemical Corporation, New Jersey) (0.5 mg/ml) at 25°C for three successive periods of 15 min each and subsequently digested with 7 ml of collagenase/dispase (dispase was obtained from Boehringer Mannheim, West Germany) (0.5 mg/ml in phosphate-buffered saline, PBS, supplementd with DNase I, 4 $\mu$g/ml) with agitation at 37°C for four successive periods of 20 min each until all tissues were completely digested. The collagenase and collagenase/dispase fractions were then layered onto a 30% FCS in PBS cushion and were allowed to sediment through this for 30 min at 4°C. The top layer (containing single cells) was aspirated and discarded and the lower layer centrifuged to recover sedimented T-ROS and TNC (Wekerle et al., 1980a). Prepared cells were plated onto 60 mm dishes and left 1 to 3 days before infection.

Virus-producing cell lines and all other cell lines used in this study were maintained in IMDM/FCS.

### Retroviral Infection and Isolation of Cell Lines

Virus stocks of the recombinant retroviruses Zip SVtsA58 and Zip E1a12S were prepared from previously characterized virus-producing $\Psi_2$ cell lines (Tegtmeyer, 1975; Mann et al., 1983; Cepko et al., 1984; Roberts et al., 1985; Jat and Sharp, 1989). Briefly, supernatants containing virus were collected from confluent flasks of virus-producing cells, filtered through 0.45-$\mu$m filters and stored at –70°C until use. Primary stromal cultures or T-ROS/TNC were infected with 2 ml of virus-containing supernatant, 1 ml polybrene (Aldrich Chemical Co. Inc.) (2 $\mu$g/ml), and 1 ml of medium for 2 hr at 37°C, and the dishes were rocked every 15 min. Complete medium was added and cells derived from an SV40tsA58 infection were transferred to 33°C and cultured for 48 hr, after which time the cells were trypsinized and split into two large petri dishes (90 mm) (Jat and Sharp, 1989). Once the cells had adhered, medium containing G418 (Davies and Jimenez, 1980) (500 $\mu$g/ml) (Gibco) was added and exchanged every 4–5 days. Colonies resistant to G418 were picked using cloning cylinders and transferred to 24 well plates and expanded.

**Reagents**

The following antibodies were used for labeling: anti-SV40 large T (PAb 412) (Harlow et al., 1981); mouse antikeratin (LE61) (Lane, 1982); polyclonal rabbit antikeratin (Sigma, Poole, U.K.); goat anti-vimentin (ICN Biomedicals, Inc., High Wycombe, U.K.); rat antimouse thymic epithelial components: ER-TR4 and ER-TR5 (van Vliet et al., 1984); rat antimouse dendritic cells: NLDL-145 and MIDC-8 (Kraak et al., 1986; Breel et al., 1987); anti-CD45 common determinant (M1/9.3) (Springer et al., 1978); anti-Thy1.1 (HO22.1) (Ledbetter and Herzenberg, 1979); anti-Thy1.2 (30-H12) (Marshak-Rothstein et al., 1979); Mac-1 (Springer et al., 1979); anti-class I (HB24, 15-5-5S; anti-$D^k$, $K^{d,f}$; mouse $IgG_{2a}$) and anticlass II antigen (Tib 120, M5/114.15.2; anti-I-$A^{b,d,q}$ and I-$E^{d,k}$; rat $IgG_{2b}$), both from ATCC; FITC conjugated swine antigoat Ig (Tago), sheep antimouse Ig (Sigma), and goat antirat Ig (Nordic Immunological Laboratories Ltd., Maidenhead, U.K.). IFNγ was obtained from Genzyme Biochemicals Ltd. (Springfield Mill, U.K.). Biotinylated Ulex europeus agglutinin (UEA) and FITC-conjugated Tetragonolobus purpureas agglutinin (TPA) were purchased from Sigma. Phycoerythrin-avidin (PE-avidin) was obtained from Biogenesis (Bournemouth, England).

**Immunofluorescence**

Isolated cells to be tested for expression of cytoplasmic antigens were grown on glass cover slips in 24 well plates. Subconfluent cultures were washed in PBS, fixed in acetone:methanol (3:1), air dried, incubated with primary antibodies for 30 min at RT, washed in PBS and incubated with FITC-conjugated second-layer antibodies for 30 min at RT. After washing (1 hr at 4°C), staining was visualized by fluorescence microscopy. Cells to be assayed for expression of surface antigens and lectin binding were stained in suspension. Cells were harvested with versene (EDTA-PBSA) washed in cold medium and kept on ice. Cells were incubated with primary antibodies or conjugated lectins for 30 min on ice, washed three times in cold buffer (PBS-A, 0.2% bovine-serum albumin), and incubated with FITC-conjugated or PE-avidin second-layer reagents. Cells were washed three times and analyzed on a FACScan (Becton Dickinson).

**Growth Kinetics of Clones at Permissive and Nonpermissive Temperature**

SV40 large T (ts) clones and E1a clones were plated in 60-mm dishes at a density of $2\times10^4$ cells/dish. Cells were allowed to attach overnight, at 33°C (ts clones) and at 37°C (E1a clones). Next day, some ts dishes were switched to 39°C and some E1a dishes to 33°C and 39°C, and cells were cultured for 8–10 days. At different time points, individual plates were trypsinized and viable cells counted.

**Cytochemical Staining**

Stromal cells were isolated by trypsinization. Clones were cytocentrifuged, air dried, and fixed in formalin vapor for 5 min and stained for nonspecific esterase using standard techniques (Gomori, 1950).

**Rosetting**

The rosetting was performed at 4°C using a cell-suspension assay. Briefly, stromal cells (harvested with versene) and unfractionated adult thymocytes (from BALB/c mice) were mixed 1:12 in a total volume of 200 μl and incubated on ice for 1 hr. The mixture was centrifuged at 200 *g* for 5 min, the pellet gently resuspended, and the number of rosettes scored using a hemocytometer.

**Electron Microscopy**

Stromal cells were grown to confluence in 60-mm petri dishes, fixed in 2.5% glutaraldehyde, post-fixed in 1% osmium tetroxide, dehydrated through graded ethanol, and embedded in resin. Sections were cut (90 mm) and visualized in a Zeiss EM10 electron microscope.

### ACKNOWLEDGMENTS

We thank Dr. Pieter Brekelmans for providing the ER-TR antibodies, Drs. Matthijs Breel and Georg Kraal for the NLDL-145 and MIDC-8 antibodies, and Dr. Zebunnisa Ikram for providing the virus-producing cell lines. We are also grateful to Ms Lindsey Goff and Professors Peter Beverley and Avrion Mitchison for helpful discussions and continued support.

*(Received November 1, 1990)*

*(Accepted March 26, 1991)*

### REFERENCES

Beardsley T.R., Piersbacher M., Wetzel G.D., and Hays E.F. (1983). Induction of T-cell maturation by a cloned line of thymic epithelium (TEPI). Proc. Natl. Acad. Sci. USA **80:** 6005–6009.

Benoist C., and Mathis D. (1989). Positive selection of the T cell repertoire: Where and when does it occur? Cell **58:** 1027–1033.

Ben-Zēev, X. (1984). Differential control of cytokeratins and vimentin synthesis by cell–cell contact and cell spreading in cultural epithelial cells. J. Cell. Biol. **99:** 1424–1433.

Berg L.J., Pullen A.M., Fazekas de St. Groth B., Mathis D., Benoist C., and Davis M.M. (1989). Antigen/MHC-specific T cells are preferentially exported from the thymus in the presence of their MHC ligand. Cell **58:** 1035–1046.

Borst J., Alexander S., Elder J., and Terhorst C. (1983). The T3 complex on human T lymphocytes involves four structurally distinct glycoproteins. J. Biol. Chem. **258:** 5135–5141.

Breel M., Mebius R.E., and Kraal G. (1987). Dendritic cells of the mouse recognised by two monoclonal antibodies. Eur. J. Immunol. **17:** 1555-1559.

Brekelmans P., and Van Ewijk W. (1990). Phenotypic characterization of murine thymic microenvironments. Seminars in Immunology **2:** 13–24.

Brightman B.K., Chandy K.G., Spencer R.H., and Fan H. (1989). A T lymphoid cell line responds to a thymic stromal cell line by expression of Thy-1 and CD4. J. Immunol. **143:** 2775–2782.

Cattermole J.A., Crosier P.S., Leung E., Overell R.W., Gillis S., and Watson, J.D. (1989). Isolation of murine fetal thymus cell lines after infection with recombinant retroviruses containing the v-*myc* and v-Ha-*ras* oncogenes. J. Immunol. **142:**. 3746–3753

Cepko C.L., Roberts B.E., and Mulligan R.C. (1984). Construction and application of a highly transmissible murine retrovirus shuttle vector. Cell **37:** 1053–1062.

Ceredig R., Lowenthal J.W., Nabholz M., and MacDonald H.R. (1985). Expression of interleukin-2 receptors as a differentiation marker on intrathymic stem cells. Nature **314:** 98–100.

Davies J., and Jimenez A. (1980). A new selective agent for enkaryotic cloning vectors. Am. J. Trop. Med. Hyg. **29** (Suppl.): 1089–1092.

Davis M.M., and Bjorkman P.J. (1988). T-cell antigen receptor genes and T-cell recognition. Nature **334:** 395–402.

Doyle C., and Strominger J.L. (1987). Interaction between CD4 and class II MHC molecules mediates cell adhesion. Nature **309:** 256–259.

Farquar M.G., and Palade G.E. (1963). Functional complexes in various epithelia. J. Cell. Biol. **17:** 375.

Farr A.G., and Anderson S.K. (1985). Epithelial heterogeneity in the murine thymus: Fucose-specific lectins bind medullary epithelial cells. J. Immunol. **134:** 2971–2977.

Farr A.G., Hosier S., Braddy S.C., Anderson S.K., Eisenhardt D.J., Yan Z.J., and Robles C.P. (1989). Medullary epithelial cell lines from murine thymus constitutively secrete IL-1 and hematopoietic growth factors and express class II antigens in response to recombinant Interferon-$\gamma$. Cell. Immunol. **119:** 427–444.

Fink P.J., Weissmann I.L., Kaplan, H.S., and Kyewski B.A. (1984). The immunocompetence of murine stromal cell-associated thymocytes. J. Immunol. **132:** 2266–2272.

Fowlkes B.J., Edison L., Mathieson B.J., and Chused T.M. (1985). Early T lymphocytes: Differentiation in vivo of adult intra-thymic precursor cells. J. Exp. Med. **162:** 802–822.

Gomori, G. (1950). An important histochemical technique for acid phosphatase. Stain Technology **31:** 189.

Harlow E., Crawford L.V., Pim D.C., and Williamson N.M. (1981). Monoclonal antibodies specific for simian virus 40 tumour antigens. J. Virol. **39:** 861–869.

Itoh T., Aizu S., Kasahara S., and Mori T. (1981). Establishment of a functioning epithelial cell line from the rat thymus. A cell line that induces the differentiation of rat bone marrow cells into T cell lineage. Biomedical. Res. **2:** 11–19.

Jat P.S., and Sharp P.A. (1989). Cell lines established by a temperature sensitive Simian virus 40 large-T antigen gene are growth restricted at the nonpermissive temperature. Mol. Cell. Biol. **9:** 1672–1681.

Jat P.S., Hoble M.D., Ataliotis P., Tanaka Y., Yannoutsos N., Larsson L., and Kiossis D. (1990). Direct derivation of conditionally immortal cell lines from H-2kb to A58 transgenic mice. Submitted for publications.

Jenkinson E.J., Kingston R., and Owen J.J.T. (1987). Importance of IL-2 receptors in intra-thymic generation of cells expressing T-cell receptors. Nature **329:** 160–161.

Kim K.H., Stellmach V., Javors J., and Fuchs E. (1987). Regulation of human mesothelial cell differentiation: Opposing roles of retinoids and epidermal growth factor in the expression of intermediate filament proteins. J. Cell. Biol. **105:** 3039–3051.

Koning F., Stingl G., Yokoyama W.M., Yamada H., Malioy W.L., Tschachler E., Shevach E.M., and Coligan J.E. (1987). Identification of a T3-associated $\alpha b$ T-cell receptor on Thy-1+ dendritic epidermal cell lines. Science **236:** 834–837.

Kosaka H., Ogata M., Hikita I., Maruo S., Sugihara S., Matsubara H., Takai Y., Hamoaka T., and Fujiwara H. (1989). Model for clonal deletion in the thymus. Proc. Natl. Acad. Sci. USA **86:** 3773–3777.

Kraak G., Breel M., Janse M., and Bruin G. (1986). Langerhans cells, veiled cells and interdigitating cells in the mouse recognised by a monoclonal antibody. J. Exp. Med. **163:** 981–997.

Kyewski B.A., Momburg F., and Schirrmacher V. (1987). Phenotype of stromal cell-associated thymocytes in situ is compatible with selection of the T cell repertoire at an "immature" stage of thymic T cell differentiation. Eur. J. Immunol. **17:** 961–967.

Kyewski B.A., Rouse R.V., and Kaplan H.S. (1982). Thymocyte rosettes: Multicellular complexes of lymphocytes and bone marrow-derived stromal cells in the mouse thymus. Proc. Natl. Acad. Sci. USA **79:** 5646–5650.

Lane E.B. (1982). Monoclonal antibodies provide specific intramolecular makers for study of epithelial tonofilament organisation. J. Cell. Biol. **92:** 665–673.

Lazarides E. (1982). Intermediate filaments: A chemically heterogenous developmentally regulated class of proteins. Ann. Rev. Biochem. **51:** 219–250.

Ledbetter J.A., and Herzenberg L.A. (1979). Xenogenic monoclonal antibodies to mouse. Immunol. Rev. **47:** 63–90.

Lo D., and Sprent J. (1986). Identity of cells that imprint H-2 restricted T cell specificity in the thymus. Nature **319:** 672–675.

Mann R., Mulligan R.C., and Baltimore D.B. (1983). Construction of a retrovirus packaging mutant and its use to produce helper-free defective retrovirus. Cell **33:** 153–159.

Marshak-Rothstein A., Fink P., Gridley G., Radlett H., Bevan M.K., and Getter M.L. (1979). Properties and applications of monoclonal antibodies directed against determinants of the Thy-1 locus. J. Immunol. **122:** 2491–2497.

Marusic-Gale S., Stephany D.A., Longo D.L., and Kruisbeek A.M. (1988). Development of $CD4^-CD8^+$ cytotoxic T-cells requires interactions with class I MHC determinants. Nature **333:** 180–183.

McKenzie I.F.C., and Potter T. (1979). Murine lymphocyte surface antigens. *In*: Advances in Immunology, Kunkel H.G., and Dixon F.J., Eds. (New York: Academic Press), p. 181.

Mizutani S., Watt S.M., Robertson D., Hussein S., Healy L.E., Furley A.J.W., and Greaves M.F. (1987). Cloning of human thymic subcapsular cortex epithelial cells with T-lymphocyte binding sites and hemopoietic growth factor activity. Proc. Natl. Acad. Sci. USA **84:** 4999-5003.

Moore M.A.S., and Owen J.J.T. (1967). Experimental studies on the development of the thymus. J. Exp. Med. **126:** 715–726.

Nakashima M., Mori K., Maeda K., Kishi H., Hirata K., Kawabuchi M., and Watanabe T. (1990). Selective elimination of double-positive immature thymocytes by a thymic epithelial cell line. Eur. J. Immunol. **20:** 47–53.

Owen J.J.T., and Ritter M.A. (1969). Tissue interaction in the development of thymus lymphocytes. J. Exp. Med. **129:** 431–442.

Palacios R., Studer S., Samaridis J., and Pelkonen J. (1989). Thymic epithelial cells induce in vitro differentiation of PRO-T lymphocyte clones into TCR$\alpha,\beta$/T3+ and TCR$\gamma,\delta$ /T3+ cells. EMBO J. **8:** 4053–4063.

Pattilo R.A., Hussa R.O., Story M.T., Ruckea A.C., Shalaby M.R., and Mattingly R.F. (1977). Tumour antigen and human chorionic gonadotropin in CaSki cells: A new epidermoid cervical cancer cell line. Science **196:** 1456–1458.

Pietrangeli C.E., Hayashi S.-I., and Kincade P.W. (1988). Stromal cell lines which support lymphocyte growth: Characterisation, sensitivity to irradiation and responsiveness to growth factors. Eur. J. Immunol. **18:** 863–872.

Potworowski E.F., Turcotte F., Beauchemin C., Hugo P., and Zelechowska M.G. (1986). Establishment and characterisation of a thymic medullary epithelial cell clone. In Vitro Cell. Dev. Biol. **22:** 557–560.

Roberts B.E., Miller J.S., Kimelman D., Cepko C.L., Lemischka I.R., and Mulligan R.C. (1985). Individual adenovirus type 5 early region 1A gene products elicit distinct alterations of cellular morphology and gene expression. J. Virol. **56:** 404–413.

Selvaraj P., Plunkett M.L., Dustin M., Sanders M.E., Shaw S., and Springer T.A. (1987). The T lymphocyte glycoprotein CD2 bind the cell surface ligand LFA-3. Nature **326:** 400–403.

Singer K.H., Harden E.A., Robertson A.L., Lobach D.F., and Haynes B.F. (1985). *In vitro* growth and phenotypic characterisation of mesodermal-derived and epithelial components of normal and abnormal human thymus. Hum. Immunol. **13:** 161–176.

Smith L. (1987). CD4+ murine T-cells develop from CD8+ precursors in vivo. Nature **326:** 798–800.

Springer T., Galfré G., Sceher D.S., and Milstein C. (1978). Monoclonal antibodies, synogenic antibodies to murine cell surface antigens: Identification of novel differentiation antigens. Eur. J. Immunol. **8:** 539–557.

Springer T., Galfré G., Secher D.S., and Milstein C. (1979). Mac-1: A macrophage differentiation antigen identified by monoclonal antibody. Eur. J. Immunol. **9:** 301–306.

Stutman, O. (1978). Intrathymic and extrathymic T cell maturation. Immunol. Rev. **42:** 138–184.

Tegtmeyer P. (1975). Function of simian virus 40 gene A in transforming infection. J. Virol. **15:** 613–618.

Tucek C.L., and Boyd R.L. (1990). Surface expression of CD4 and Thy-1 on thymic stromal cells. Int. Immunol. **2:** 593–601.

van Ewijk W., Jenkinson E.J., and Owen J.J.T. (1982). Detection of Thy-1, T-200, Lyt-1 and Lyt-2 bearing cells in the developing lymphoid organs of the mouse embryo *in vivo* and *in vitro*. Eur. J. Immunol. **12:** 262–271.

van Vliet E., Melis M., and van Ewijk W. (1984). Monoclonal antibodies to stromal cell types of the mouse thymus. Eur. J. Immunol. **14:** 524–529.

Vollger L.W., Tuck D.T., Springer T.A., Haynes B.F., and Singer K.H. (1987). Thymocyte binding to human epithelial cells is inhibited by monoclonal antibodies to CD-2 and LFA-3 antigens. J. Immunol. **138:** 358–363.

von Boehmer H., and Hafen K. (1986). Minor but not major histocompatibility antigens of thymus epithelium tolerize precursors of cytolytic T-cells. Nature **320:** 626–628.

Wekerle H., Ketelsen U.-P., and Ernst M. (1980a). Thymic nurse cells. Lymphoepithelial cell complexes in murine thymuses: Morphological and serological characterisation. J. Exp. Med. **151:** 925–944.

Wekerle H., Ketelsen U.-P., and Ernst M. (1980b). Thymic nurse cells. Ia bearing epithelium involved in T-lymphocyte differentiation. Nature **283:** 402–404.

Zinkernagel R.M. (1982). Selection of restriction specificities of virus-specific cytotoxic T cells in the thymus: No evidence for a crucial role of antigen presenting cells. J. Exp. Med. **156:** 1842–1847.

